# Effects of the COVID-19 Pandemic on Incidence and Epidemiology of Catheter-Related Bacteremia, Spain

**DOI:** 10.3201/eid2811.220547

**Published:** 2022-11

**Authors:** Oriol Gasch, Laia Badia-Cebada, Joao Carmezim, Montserrat Vaqué, Virginia Pomar, Encarna Moreno, Anna Marrón, Emili Jiménez-Martínez, Maria José García-Quesada, Xavier Garcia-Alarcón, Dolors Domènech, Jordi Càmara, Marta Andrés, Judith Peñafiel, Rosario Porrón, Enric Limón, Esther Calbo, Miquel Pujol

**Affiliations:** Universitat Autònoma de Barcelona, Barcelona, Spain (O. Gasch);; Hospital Universitari Parc Taulí I3PT, Sabadell, Spain (O. Gasch, L. Badia-Cebada, A. Marrón);; Institut d'Investigació Biomèdica de Bellvitge-IDIBELL, L'Hospitalet de Llobregat, Spain (J. Carmezim, J. Càmara, J. Peñafiel, M. Pujol);; Hospital de Barcelona, Barcelona (M. Vaqué);; Hospital de la Santa Creu i Sant Pau, Barcelona (V. Pomar);; Parc Sanitari Sant Joan de Déu, Sant Boi de Llobregat, Spain (E. Moreno);; Hospital Universitari de Bellvitge, L'Hospitalet de Llobregat (E. Jiménez-Martínez, J. Càmara, M. Pujol);; Hospital Germans Trias i Pujol, Badalona, Spain (M.J. García-Quesada);; Hospital Josep Trueta, Girona, Spain (X. Garcia-Alarcón, D. Domènech);; Hospital Consorci de Terrassa, Terrassa, Spain (M. Andrés);; VINCat, Barcelona (R. Porrón, E. Limón);; Universitat de Barcelona, Barcelona (E. Limón);; Hospital Universitari Mútua Terrassa, Terrassa (E. Calbo)

**Keywords:** COVID-19, respiratory infections, severe acute respiratory syndrome coronavirus 2, SARS-CoV-2, SARS, coronavirus disease, zoonoses, viruses, coronavirus, catheter-related bacteremia, quality of care, patient safety, Catalonia, Spain, bacteria

## Abstract

We compared hospital-acquired catheter-related bacteremia (CRB) episodes diagnosed at acute care hospitals in Catalonia, Spain, during the COVID-19 pandemic in 2020 with those detected during 2007–2019. We compared the annual observed and predicted CRB rates by using the negative binomial regression model and calculated stratified annual root mean squared errors. A total of 10,030 episodes were diagnosed during 2007–2020. During 2020, the observed CRB incidence rate was 0.29/10^3^ patient-days, whereas the predicted CRB rate was 0.14/10^3^ patient-days. The root mean squared error was 0.153. Thus, a substantial increase in hospital-acquired CRB cases was observed during the COVID-19 pandemic in 2020 compared with the rate predicted from 2007–2019. The incidence rate was expected to increase by 1.07 (95% CI 1–1.15) for every 1,000 COVID-19–related hospital admissions. We recommend maintaining all CRB prevention efforts regardless of the coexistence of other challenges, such as the COVID-19 pandemic.

In December 2019, the first cases of COVID-19 were reported in Wuhan, China (*1*). On March 11, 2020, the World Health Organization declared COVID-19 a global pandemic because of the spread of SARS-CoV-2 infections worldwide (*2*). Subsequent waves related to the spread of different SARS-CoV-2 serotypes forced healthcare systems and, specifically, acute care hospitals to modify their structural and human resource organization (*3*); scheduled elective surgeries were cancelled, and healthcare workers had to change their specific clinical roles to address the abrupt increase in admissions of SARS-CoV-2–infected patients. To reduce SARS-CoV-2 nosocomial transmission, airborne and contact precaution measures were reinforced, personal protective equipment was worn by healthcare providers, and strict hand hygiene measures were observed at most centers (*4*).

Hand hygiene is a cornerstone of healthcare-associated infection (HAI) prevention, and reductions in *Clostridioides difficile* colitis incidence (*5*,*6*) and surgical-site infections (*7*,*8*) have been observed in different settings during the COVID-19 pandemic. However, reductions in other HAIs, such as catheter-associated urinary tract infections, ventilator-associated pneumonia, or catheter-related bacteremia (CRB) (*9*,*10*), were not observed. In addition, multidrug-resistant microorganisms were increasingly involved in these other HAIs (*10*–*12*).

CRB is one of the most frequent HAIs (*13*,*14*) and represents a major health challenge because of its high association with illness and death (*15*,*16*). CRB is currently considered a leading safety concern in healthcare settings and is a clinical practice quality indicator (*17*). For these reasons, CRB surveillance is mandatory in most countries (*18*–*20*).

In Catalonia, Spain, CRB surveillance is guided by the VINCat program of the Catalan Health Service (*21*), which provides a surveillance system for healthcare-associated nosocomial infections. The VINCat program was launched in 2006; the main objective of this program is to reduce the incidence of HAIs through continuous active monitoring and implementation of preventive programs (*21*). During recent decades, the incidence of healthcare-acquired CRB has decreased in most hospitals, especially in intensive care units (ICUs), because of the application of preventive measures (*22*,*23*). Some of the most critical evidence-based preventive interventions have been using appropriate barrier precautions and hand hygiene before handling catheters, disinfecting skin with chlorhexidine solutions, using appropriate catheter materials, carefully selecting insertion sites that avoid the femoral site, and withdrawing catheters whenever possible (*24*). During the COVID-19 pandemic, adherence to some of these preventive measures has notably affected HAI incidence rates (*11*); however, the effect of COVID-19 on CRB incidence is not definitively known. The aim of this study was to assess the effects of the COVID-19 pandemic on the incidence of hospital-acquired CRB.

## Materials and Methods

### Clinical Setting

Bacteremia associated with the use of venous catheters was continuously monitored under the VINCat program. All nosocomial episodes of CRB diagnosed in adult patients at each participating hospital were prospectively followed and reported to the VINCat program by infection control teams. CRB cases were identified by daily evaluation of all patients with bacteria-positive blood cultures.

Hospitals participating in the VINCat program are classified into 3 categories according to the number of beds available for hospitalization: >500 beds (group I), 200–499 beds (group II), and <200 beds (group III). Data from each hospital are continuously monitored and presented in general clinical sessions. A public annual report is published on the VINCat website (*21*).

### Definitions

We defined catheter-related bacteremia as the detection of bacterial growth in patient blood using a venous catheter; >1 set of blood cultures were obtained from a peripheral vein and 2 sets were obtained to identify habitual skin-colonizing microorganisms, such as coagulase-negative staphylococci, *Micrococcus* spp., *Propionibacterium acnes*, *Bacillus* spp., and *Corynebacterium* spp. Positive bacterial cultures had to be associated with clinical manifestations of infection, such as fever, chills, or hypotension, and absence of any apparent alternative source of bloodstream infection (BSI). The conditions had to be accompanied by >1 of the following criteria: >15 CFU per catheter segment in semiquantitative cultures or >10^3^ CFU per catheter segment in quantitative cultures that detected the same microorganism found in peripheral blood cultures; quantitative blood cultures that detected the same microorganism and showed a difference of >5:1 between the blood obtained from the lumen of a venous catheter and that obtained from a peripheral vein by puncture; difference of >2 hours between positive bacterial cultures obtained from a peripheral vein and the lumen of a venous catheter; presence of inflammatory signs or purulent secretions in the insertion point or the subcutaneous tunnel of a venous catheter (a culture of the secretion showing growth of the same microorganism detected in the blood cultures was also useful); and resolution of clinical signs and symptoms after catheter withdrawal with or without appropriate antibiotic treatment. For the clinical diagnosis of peripheral venous CRB, we required signs of phlebitis (induration, pain, or signs of inflammation at the insertion point or the catheter route).

### Exclusion Criteria

We excluded patients if they were under 18 years of age, were outpatients, and had a hospital stay <48 hours at the time of BSI detection. We also excluded those who had CRB detected at an outpatient service or had CRB associated with arterial catheters.

### Microbiology

Two sets of 2 blood samples from a peripheral vein were obtained from all patients with a suspected BSI. An additional blood sample was also obtained through the catheter. When possible, the catheter tip was cultured after removal. Blood samples were processed at the microbiology laboratories of each center in accordance with standard operating procedures. All microorganisms were identified by using standard microbiological techniques at each center.

### Statistical Analysis

We reported categorical variables as the number of cases and percentages and continuous variables as means +SD or medians with interquartile ranges, depending on whether the distribution was normal or nonnormal. We assessed normality of variables graphically by using quantile-quantile and density plots. We calculated the CRB incidence rate by dividing the total number of episodes of CRB by the total number of hospital stays (patient-days) in 1 year.

We used a negative binomial regression model to assess the rate trend of CRBs diagnosed at VINCat hospitals each year during 2007–2019. We used the number of admissions per year as the offset variable, number of events as the dependent variable, and year as the main independent variable. We performed stratified analyses according to hospital ward, catheter type, catheter insertion site, catheter use, and type of identified microorganism. We reported the annual rate of CRBs diagnosed per 1,000 patient-days and the incidence rate ratio (IRR) and 95% CI for each model. We focused the interpretation of the IRR on the annual rate of increase or decrease.

We plotted and compared the annual CRB rates observed during 2007–2020 and annual CRB rates predicted by our model. We calculated the average root mean squared error (RMSE) of the model predictions for CRB rates during 2007–2019 and compared the RMSEs between the expected rate according to the model and observed rate in 2020. We replicated these analyses after stratifying by hospital ward, catheter type, catheter insertion site, catheter use, and type of microorganism.

We evaluated the conditions of application in all models and calculated the 95% CI for each estimator. We arbitrarily set the level of statistical significance at 5%. We performed the analyses using the statistical package R version 4.0.3 (The R Project for Statistical Computing, https://www.r-project.org) for Windows.

### Ethical Considerations

Participation in the VINCat program was voluntary, and data confidentiality was guaranteed. This study was evaluated and approved by the Parc Taulí Hospital Research Ethics Committee, Sabadell, Spain.

## Results

### Study Periods

During 2007–2020, a total of 10,030 nosocomial episodes of CRB were diagnosed. Data from the 2007–2019 period have been analyzed and described previously (*25*). In summary, during 2007–2019, a total of 9,290 episodes of CRB were diagnosed. The mean annual incidence was 0.2 episodes/10^3^ patient-days, 73.7% of episodes occurred in non-ICU wards, 62.7% of episodes were related to central vascular catheters, 24.1% of episodes were related to peripheral venous catheters, and 13.3% of episodes were related to peripherally inserted central venous catheters (*25*). The incidence rate of CRB decreased substantially over the 2007–2019 study period (IRR 0.94, 95% CI 0.93–0.96), especially in ICU wards. CRB episodes caused by central vascular catheters fell markedly (IRR 0.90, 95% CI 0.89–0.92), whereas those associated with peripherally inserted catheters increased.

**Figure 1:**
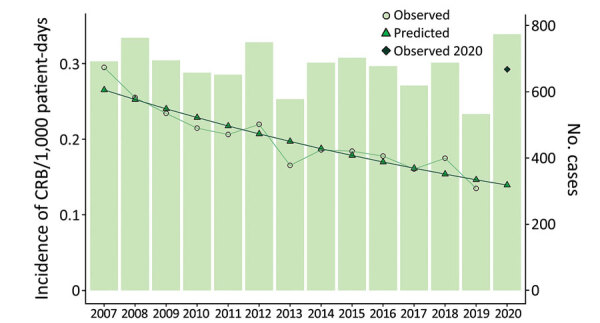
Observed and predicted incidence rates of CRB during 2007–2020 in study of effects of the COVID-19 pandemic on incidence and epidemiology of CRB, Spain. We calculated the CRB incidence rate by dividing the total number of episodes of catheter-related bloodstream infections by the total number of hospital stays (patient-days) for each year from 2007 to 2020. We predicted incidence rates by using the negative binomial regression model and compared the predicted rates with observed rates for each year. CRB, catheter-related bacteremia.

In 2020, a total of 774 CRB episodes were diagnosed at the participating hospitals. We determined that the incidence rate was 0.29 episodes/10^3^ patient-days (Figure 1). Of 774 episodes, 297 (40.1%) were acquired in conventional medical wards, 127 (17.2%) in surgical wards, and 316 (42.7%) in ICUs. We found that the catheters most frequently implicated in CRB were central venous catheters (412 cases, 55.7%), peripheral catheters (169 cases, 22.8%), and peripherally inserted central venous catheters (159 cases, 21.5%). Catheters causing CRB were located in the arm/forearm (323 cases, 43.6%), jugular (237 cases, 32.0%), subclavian (116 cases, 15.7%), or femoral (52 cases, 7.03%) sites. The catheters were used for medication and serum infusion (583 cases, 78.8%), parenteral nutrition (146 cases, 19.7%), or hemodialysis (11 cases, 1.5%). The most frequent causes of CRB were coagulase-negative staphylococci (299 cases, 41.3%), *Staphylococcus aureus* (155 cases, 21.4%), gram-negative enterobacteria (112 cases, 15.5%), enterococci (72 cases, 9.9%), *Candida* sp*.* (45 cases, 6.2%), and *Pseudomonas aeruginosa* (34 cases, 4.7%).

### Comparison of Observed and Expected Incidence Rates

According to the case mix index observed during 2007–2019, we predicted that the incidence rate for CRB in 2020 was 0.14 episodes/10^3^ patient-days. However, we observed 0.29 episodes/10^3^ patient-days (observed/predicted [O/P] ratio 2.10, 95% CI 1.95–2.25) in 2020. The RMSE was 0.015 during 2007–2019 and 0.153 in 2020 (Figure 1). Disparities between predicted and observed rates were consistent among the different participating hospitals (Appendix Figure 1). 

**Figure 2:**
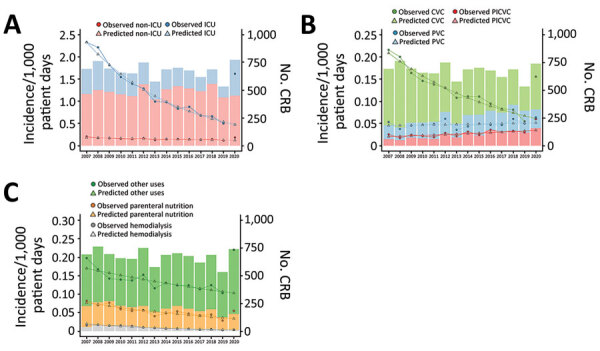
Observed and predicted incidence rates of CRB and number of CRB cases stratified by hospital ward, catheter type, and catheter use during 2007–2020 in study of effects of the COVID-19 pandemic on incidence and epidemiology of CRB, Spain. We calculated the CRB incidence rate by dividing the total number of episodes of catheter-related bloodstream infections by the total number of patient-days for each year from 2007 to 2020. We predicted incidence rates by using the negative binomial regression model and compared the predicted rates with observed rates for each year. A) CRB incidence per 1,000 patient-days, stratified by the type of hospital ward. B) CRB incidence per 1,000 patient-days, stratified by the type of catheter used. C) CRB incidence per 1,000 patient-days was stratified according to the reason for catheter use. CRB, catheter-related bacteremia; ICU, intensive care unit; CVC, central vascular catheter; PICVC, peripherally-inserted central vascular catheter; PVC, peripheral vascular catheter; PN, parenteral nutrition; HD, hemodialysis.

In conventional surgical and medical wards, we found that the predicted incidence rate for CRB was 0.12 episodes/10^3^ patient-days, and the observed rate was 0.19/10^3^ patient-days in 2020 (O/P 1.51, 95% CI 1.37–1.65). However, in ICUs, we predicted the incidence rate was 0.48 episodes/10^3^ patient-days, but the observed rate was 1.62/10^3^ patient-days in 2020 (O/P 3.42, 95% CI 3.04–3.79). The average RMSE was 0.013 for conventional wards and 0.069 for ICUs during 2007–2019, whereas, in 2020, the RMSE was 0.062 for conventional wards and 1.147 for ICUs (Table 1; Figure 2).

**Table 1:** Incidence rates of CRB per 1,000 patient-days in 2020 stratified by catheter characteristics and microorganisms in study of effects of the COVID-19 pandemic on incidence and epidemiology of catheter-related bacteremia, Spain*

Category	Observed rate	Predicted rate	Observed/predicted (95% CI)	RMSE
Location acquired				
ICU	1.62	0.48	3.42 (3.04–3.79)	1.147
Non-ICU	0.19	0.12	1.51 (1.37–1.65)	0.062
Catheter type				
CVC	0.16	0.06	2.54 (2.29–2.78)	0.094
PICVC	0.06	0.04	1.73 (1.46–2.00)	0.025
PVC	0.06	0.05	1.24 (1.06–1.43)	0.012
Catheter insertion site				
Arm/forearm	0.12	0.08	1.45 (1.29–1.60)	0.038
Jugular	0.09	0.03	2.64 (2.30–2.97)	0.056
Subclavian	0.04	0.02	1.88 (1.53–2.22)	0.020
Femoral	0.02	0.01	3.12 (2.27–3.96)	0.013
Catheter use				
Serum/medication	0.22	0.10	2.14 (1.97–2.31)	0.117
Hemodialysis	0.00	0.00	1.25 (0.51–1.99)	0.001
Parenteral nutrition	0.06	0.03	1.62 (1.36–1.89)	0.021
Microorganism				
* Staphylococcus aureus*	0.06	0.04	1.26 (1.06–1.46)	0.012
Coagulase-negative staphylococci	0.11	0.05	2.41 (2.14–2.68)	0.066
Gram-negative bacteria	0.04	0.03	1.69 (1.38–2.01)	0.017
*Enterococcus* sp.	0.03	0.01	5.41 (4.16–6.65)	0.022
* Pseudomonas aeruginosa*	0.01	0.01	2.20 (1.46–2.94)	0.007
*Candida* sp*.*	0.02	0.01	2.24 (1.59–2.90)	0.009

*We predicted expected rates of CRB by using negative binomial regression models and compared predicted rates with observed CRB rates in 2020. CRB, catheter-related bacteremia; RMSE, root mean squared error; ICU, intensive care unit; CVC, central vascular catheter; PICVC, peripherally inserted central vascular catheter; PVC, peripheral vascular catheter.

We observed an incidence rate of 0.064 for CRB caused by peripheral catheters in 2020; the predicted rate according to the negative binomial regression model was 0.05 (O/P 1.24, 95% CI 1.06–1.43). When central catheters were used, the observed rate for CRB was 0.16, and the predicted rate was 0.06 (O/P 2.54, 95% CI 2.29–2.78). When peripherally inserted central catheters were used, the observed rate for CRB was 0.06, and the predicted rate was 0.04 (O/P 1.73, 95% CI 1.46–2.00). We observed increases in RMSEs in 2020 compared with the 2007–2019 period for peripheral catheters (0.012 vs. 0.007), central catheters (0.094 vs. 0.008), and peripherally inserted central catheters (0.025 vs. 0.004) (Table 1; Figure 2). In addition, we determined that the number of observed CRB episodes in 2020 were higher than predicted episodes depending on the location of the catheter; increased incidence was more pronounced in catheters located in femoral (O/P 3.11, 95% CI 2.27–3.96), jugular (O/P 2.64, 95% CI 2.30–2.97), and subclavian (O/P 1.88, 95% CI 1.53–2.22) sites (Table 1; Appendix Figure 2).

In 2020, we found increases in observed CRB incidence rates compared with rates predicted by the binomial regression model according to catheter use and causative microorganisms. For hemodialysis, the observed CRB rate was 0.004, and the predicted rate was 0.003 (O/P 1.25, 95% CI 0.51–1.99). For parenteral nutrition, the observed CRB rate was 0.06, and the predicted rate was 0.03 (O/P 1.62, 95% CI 1.36–1.89). For other uses, the observed CRB rate was 0.22, and the predicted rate was 0.10 (O/P 2.14, 95% CI 1.97–2.31); the last category increased most notably (Table 1; Figure 2). Observed CRB rates were increased compared with predicted rates for all causative microorganisms, especially enterococci (O/P 5.41, 95% CI 4.16–6.65).

### Relationship between Monthly CRB Incidence Rates and SARS-CoV-2 Admissions

**Figure 3:**
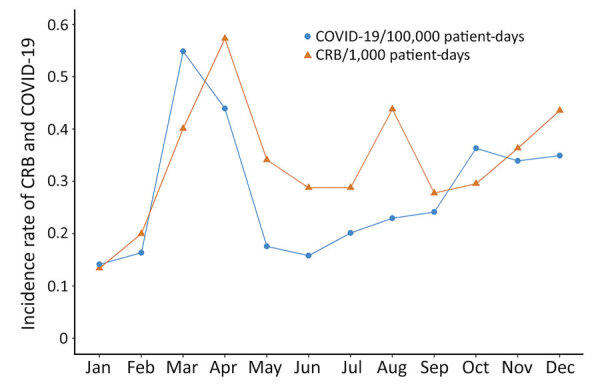
COVID-19–related hospital admissions and CRB incidence rates in 2020 in study of effects of the COVID-19 pandemic on incidence and epidemiology of CRB, Spain. We compared the incidence rates for COVID-19-related hospital admissions with rates for CRB each month during 2020. We calculated the COVID-19 incidence rates by dividing the total number of COVID-19 admissions by the total number of patient-days. We calculated CRB incidence rates by dividing the total number of episodes of catheter-related bloodstream infections by the total number of patient-days. CRB, catheter-related bacteremia.

The total number of hospital admissions and the proportion of patients affected by COVID-19 changed substantially during 2020 (Figure 3). We recorded more COVID-19–related admissions during February–June in both conventional wards and ICUs (Table 2; Figure 3). The peak rate of COVID-19 hospital admissions was 54.87 in March, and the lowest rate was 14.15 in January.

**Table 2:** Temporal evolution of COVID-19–related hospital admissions and catheter-related bacteremia incidence rates in study of effects of the COVID-19 pandemic on incidence and epidemiology of catheter-related bacteremia, Spain, 2020

Month	Conventional ward			ICU			Total*	
	Rate of COVID-19 admissions†	CRB incidence rate‡		Rate of COVID-19 admissions†	CRB incidence rate‡		Rate of COVID-19 admissions†	CRB incidence rate‡
January	14.11	0.12		15.15	1.39		14.15	0.13
February	16.12	0.15		21.78	2.86		16.36	0.20
March	52.81	0.13		76.14	3.29		54.87	0.40
April	41.30	0.20		83.44	5.55		43.95	0.57
May	17.02	0.22		31.33	4.49		17.6	0.34
June	15.56	0.21		21.37	2.81		15.8	0.29
July	19.50	0.17		32.94	2.83		20.15	0.29
August	22.10	0.24		38.41	5.73		22.96	0.44
September	23.06	0.18		46.00	3.43		24.15	0.28
October	34.20	0.14		70.45	3.20		36.33	0.30
November	31.87	0.20		68.22	4.09		33.93	0.36
December	33.73	0.29		68.43	7.11		34.94	0.44

*Total rates for conventional plus ICU wards. ICU, intensive care unit; CRB, catheter-related bacteremia. †Values are COVID-19 admission rates per 100,000 patient-days. ‡Values are episodes of CRB per 1,000 patient-days.

Concomitantly, CRB incidence rates also varied during 2020, reaching a peak in April (0.57 episodes of CRB/10^3^ admissions), followed by August and December (0.44 episodes of CRB/10^3^ admissions for each month) (Table 2). We observed the lowest CRB rate at the beginning of the year (0.13 episodes of CRB/10^3^ admissions). 

**Figure 4:**
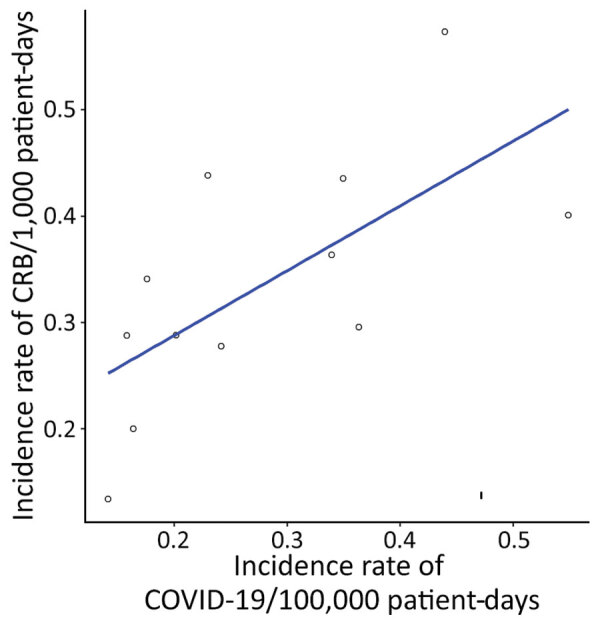
Association between COVID-19–related hospital admissions and CRB incidence rate in 2020 in study of effects of the COVID-19 pandemic on incidence and epidemiology of CRB, Spain. We calculated COVID-19 incidence rates by dividing the total number of COVID-19 admissions by the total number of patient-days and CRB incidence rates by dividing the total number of episodes of catheter-related bloodstream infections by the total number of patient-days. We used linear regression analysis to determine the relationship between COVID-19–related hospital admissions and the incidence of CRB. We found a positive association between the incidence of COVID-19–related hospital admissions and incidence rate of CRB (R^2^ = 0.45). CRB, catheter-related bacteremia.

We observed an association between CRB and COVID-19 incidence rate. The CRB incidence rate was expected to increase by 1.07 (IRR 1.07, 95% CI 1–1.15) for every 1,000 COVID-19 admissions if all factors remained constant (Figure 4).

## Discussion

We demonstrated that the COVID-19 pandemic increased CRB incidence in 2020 in our hospitals in Catalonia, Spain. We found that months with the highest proportion of COVID-19 admissions were strongly associated with increased CRB incidence. We also described the most critical CRB characteristics that changed during the pandemic in 2020. Compared with previous years, we observed increased CRB incidence in both ICUs and conventional wards in 2020.

Other studies conducted around the same time observed increased HAI incidence rates during 2020, especially in ICUs. Catheter-associated urinary tract infections, ventilator-associated pneumonia, and CRB were the HAIs with the greatest increases (*9*–*11*). In contrast, other HAIs, such as nosocomial-acquired *C. difficile* colitis (*5*,*6*) or surgical-site infections (*7*,*8*, decreased during the same period. Of note, HAIs may be more frequently associated with patients receiving steroids or tocilizumab (*26*), although a specific association with BSI was not observed (*27*).

In most cases, the increased rates of CRB were likely associated with a lower adherence to specific preventive measures during the months when the pandemic caused the most hospital admissions, despite the generalized reinforcement of contact precautions and hand hygiene to reduce SARS-CoV-2 nosocomial transmission. Of note, in our hospital settings, alcohol-based product consumption for hand hygiene during 2020 increased 2.4-fold overall and 1.9-fold in ICUs compared with the previous year, and a similar trend was observed in a hospital in Taiwan (*28*). Therefore, although proper hand hygiene is necessary to prevent CRB and other HAIs, it is not sufficient to avoid HAIs if other measures are not performed during the insertion and care of vascular catheters. Specifically, since 2006, various evidence-based bundles for CRB interventions have been shown to reduce CRB, especially in the ICU setting. These bundles include handwashing, using full-barrier precautions, cleaning the skin with chlorhexidine, avoiding the femoral site if possible, and removing unnecessary catheters (*22*,*23*). Among the different preventive measures, both hand hygiene and catheter insertion measures were associated with reduced incidence of CRB, and they were most effective when both measures were applied simultaneously (*24*).

The first limitation of our study is that heterogeneity of COVID-19 pandemic responses existed between hospitals, resulting in lack of data on adherence to CRB preventive measures at each center. Second, there was a lack of clinical information regarding the presence of chronic diseases or clinical conditions that might influence CRB incidence rates. However, the availability of a large number of CRB episodes diagnosed by standardized definitions is a strength that enables generalization of our observations. In addition, CRB incidence rates were adjusted by patient-days rather than catheter-days, which enabled surveillance of all types of catheters inserted in all hospital wards. 

In 2020, substantial resources were allocated for infection prevention to manage the SARS-CoV-2 outbreak, which also affected HAI prevention programs. Because CRB is a key healthcare quality indicator (*29*), our observations stress the importance of maintaining all prevention efforts, regardless of the coexistence of other challenges, such as the worldwide COVID-19 pandemic.

Appendix Additional information on effects of the COVID-19 pandemic on incidence and epidemiology of catheter-related bacteremia, Spain.
