# Supplementary material for: Effects of the COVID-19 Pandemic on Incidence and Epidemiology of Catheter-Related Bacteremia, Spain
Source: Emerg Infect Dis. 2022 Nov;28(11):2181–9. doi: 10.3201/eid2811.220547 (PMC9622263; doi:10.3201/eid2811.220547)
Supplement: Appendix — Additional information on effects of the COVID-19 pandemic on incidence and epidemiology of catheter-related bacteremia, Spain. [file 22-0547-Techapp-s1.pdf]

Article DOI: https://doi.org/10.3201/eid2811.220547

Effects of the COVID-19 Pandemic on Incidence and Epidemiology of Catheter-Related Bacteremia, Spain

Appendix

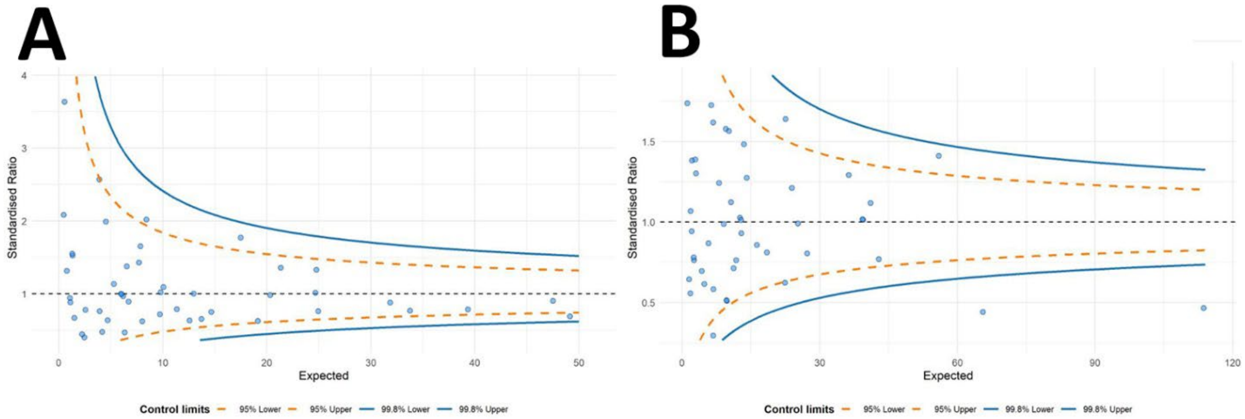

Appendix Figure 1. Comparison of the observed/predicted rates of catheter-related bacteremia in participating hospitals in study of effects of the COVID-19 pandemic on incidence and epidemiology of catheter-related bacteremia, Spain. Funnel plots show the expected variation in observed/predicted rates of catheter-related bacteremia in 2019 (A) compared with 2020 (B) using a standardized ratio of 1.0. Lines indicate the 95% and 99.8% upper and lower confidence (control) limits. In 2020, two hospitals had an observed/predicted bacteremia ratio below the 99.8% confidence limit.

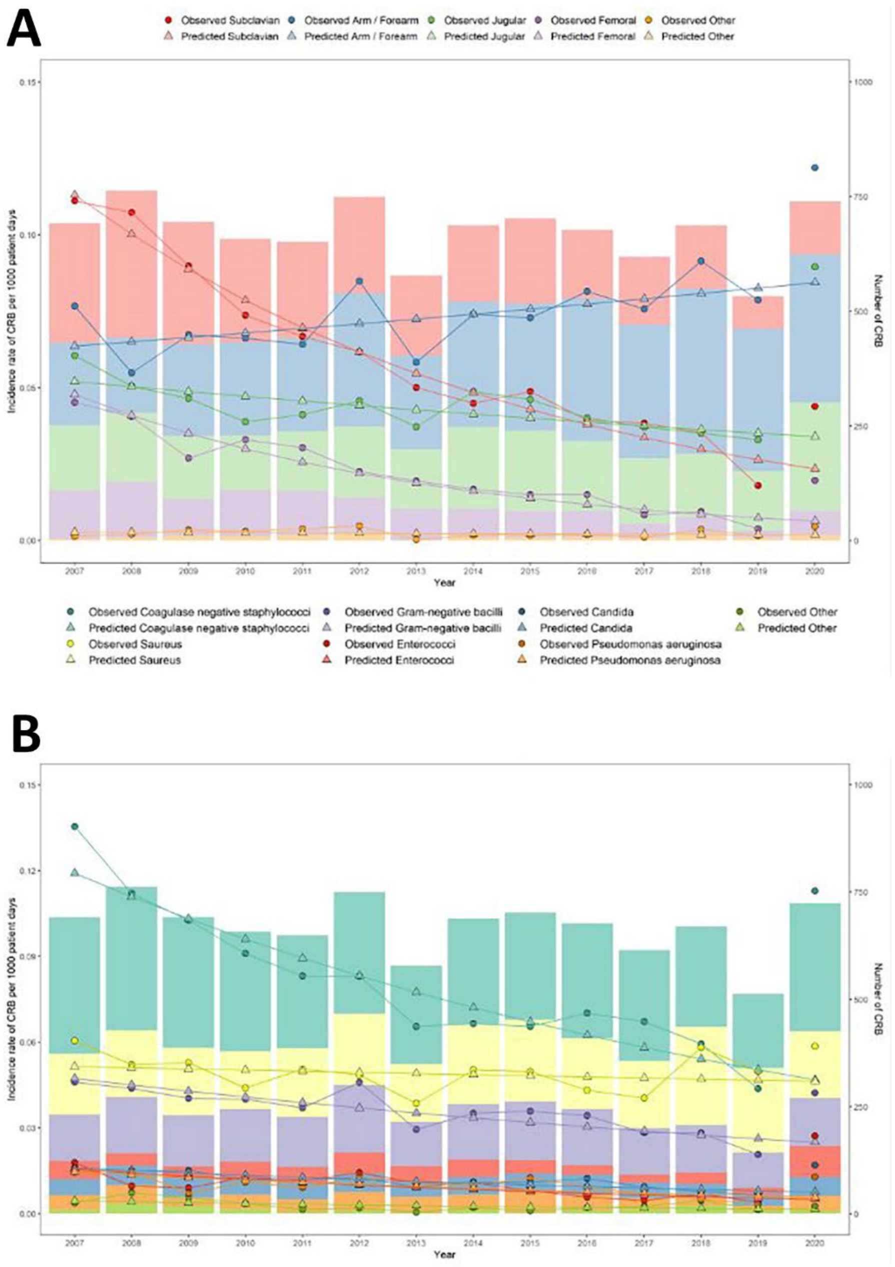

Appendix Figure 2. Observed and predicted incidence rates for CRB and number of CRB cases stratified by catheter location and microorganism during 2007–2020 in study of effects of the COVID-19 pandemic on incidence and epidemiology of catheter-related bacteremia, Spain. We calculated the CRB incidence rate by dividing the total number of episodes of catheter-related bloodstream infections by the total number of patient-days for each year during 2007–2020. We predicted incidence rates by using the negative binomial regression model and compared the predicted rates with observed rates for each year. A) CRB incidence per 1,000 patient-days was stratified by the physical location of the catheters. B) CRB incidence per 1,000 patient-days was stratified by the type of microorganisms responsible for the bacteremia. CRB, catheter-related bacteremia.
